# Characterization of bacterial diversity in rhizospheric soils, irrigation water, and lettuce crops in municipalities near the Bogotá river, Colombia

**DOI:** 10.1016/j.heliyon.2024.e35909

**Published:** 2024-08-06

**Authors:** Rodrigo A. Echeverry-Gallego, Diana Martínez-Pachón, Nelson Enrique Arenas, Diego C Franco, Alejandro Moncayo-Lasso, Javier Vanegas

**Affiliations:** a Grupo de Investigación en Ciencias Biológicas y Químicas, Facultad de Ciencias, Universidad Antonio Nariño, Bogotá DC, Colombia; b Doctorado en Ciencia Aplicada, Universidad Antonio Nariño, Bogotá DC, Colombia; c Facultad de Medicina, Universidad de Cartagena. Cartagena, Colombia; d Institute of Environmental Sciences, Faculty of Biology, Jagiellonian University, Krakow, Poland

**Keywords:** Foodborne diseases, Wastewater, Food safety, Endophytes, Sanitation

## Abstract

The use of wastewater in agricultural practices poses a potential risk for the spread of foodborne diseases. Therefore, this study aimed to characterize the bacterial biodiversity in rhizospheric soil, irrigation water, and lettuce crops in three municipalities adjacent to the Bogotá River, Colombia. Samples were collected in Mosquera, Funza, and Cota municipalities, including rhizospheric soil, lettuce leaves, and irrigation water. The total DNA extraction was performed to analyze bacterial diversity through high-throughput sequencing of the 16S ribosomal RNA genes, utilizing the Illumina HiSeq 2500 PE 300 sequencing platform. A total of 198 genera from the rhizospheric soil were detected including a higher abundance of zOTUs such as *Bacillus*, *Streptomyces*, and clinically relevant genera such as *Mycobacterium* and *Pseudomonas*. In lettuce, the detection of 26 genera of endophytic bacteria showed to Proteobacteria and Firmicutes as the predominant phyla, with *Staphylococcus* and *Bacillus* as the most abundant genera. Notably, Funza's crops exhibited the highest abundance of endophytes, approximately 50 %, compared to Cota (20 %). Furthermore, the most abundant bacterial genera in the irrigation water were *Flavobacterium* and *Pseudomonas*. The most prevalent Enterobacteriaceae were *Serratia, Enterobacter, Citrobacter, Klebsiella, Yersinia, Shigella, Escherichia,* and *Erwinia*. The *Bacillus* genus was highly enriched in both rhizospheric soils and lettuce crops, indicating its significant contribution as the main endophytic bacterium.

## Highlights

• Bacterial biodiversity in rhizospheric soil, irrigation water, and lettuce crops was characterized.
• Potentially pathogenic genera were identified in the rhizosphere, irrigation water, and lettuce endophytes.
• No relationship in microbial composition was detected among the samples studied.
• Proteobacteria, manly Enterobacterales, dominated endophytic, but their presence does not imply pathogenicity.

## Graphical abstract

**Figure:**
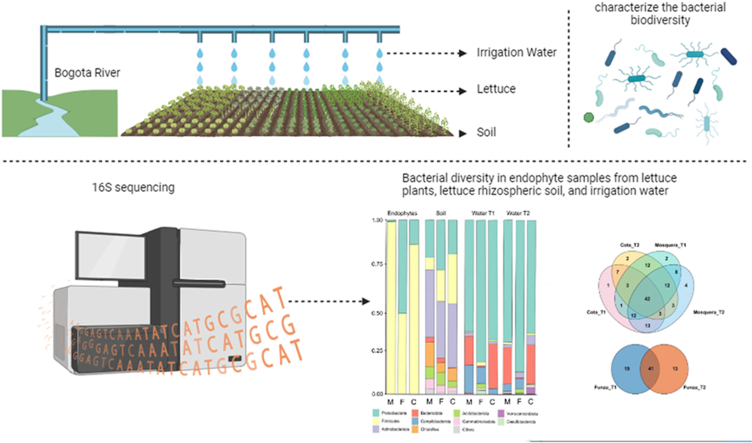

## 1 Introduction

Lettuce (*Lactuca sativa*) is a widely consumed vegetable, used by millions of people worldwide [1]. It plays a vital role in the agricultural sector of various countries, such as the United States (4.2 million tons), Mexico (0.54 million tons), and Colombia (0.21 million tons) [2]. In Colombia, Cundinamarca is the primary region for lettuce production, contributing around 49 336 tons and generating an estimated income of USD 106 000. Significant yields are observed in the municipalities of Mosquera, Madrid, Funza, and Cota [3,4]. Lettuce cultivation in these areas heavily relies on a consistent water supply, which is sometimes obtained from polluted sources containing a wide range of Pharmaceutically Active Compounds (PhACs) and hosting numerous human pathogens [5].

Despite various cooking strategies aimed at avoiding bacterial contamination in food, disease outbreaks still occur in many countries, with leafy fresh vegetables posing a significant risk [6]. Lettuce, being the most widely consumed leafy vegetable globally and often consumed raw, increases the potential for foodborne outbreaks. The World Health Organization estimates an alarming 420–960 million cases of foodborne illnesses annually, resulting in approximately 420 000 deaths and causing significant disability and high medical expenses among healthy populations [7]. Notably, *Salmonella* spp., *Campylobacter*, enterohaemorrhagic *Escherichia coli*, and *Listeria* have been isolated as the most common causative agents in such cases [8]. For instance, in Sweden, the consumption of lettuce irrigated with crop wastewater has been linked to foodborne illnesses caused by *E. coli* O157:H7 [9]. Similarly, in the US and Canada, an outbreak of *E. coli* O157:H7 resulted in 62 cases and 25 hospitalizations due to the consumption of lettuce and cauliflower irrigated with contaminated water [10]). Similarly, Colombian national health authorities have reported that foodborne outbreaks with pathogen identification were only assessed in 31,2 % of total cases and mostly associated with *E. coli, S. aureus, Salmonella* spp. and coliforms in 2021 [11]. Moreover, these pathogens have been reported as endophytic colonizers of agricultural plants, indicating that despite thorough washing, they can remain inactive and potentially trigger outbreaks of foodborne diseases [12].

The middle basin of the Bogotá River in Colombia is known for its high levels of microbiological contamination [13,14], the presence of human pathogens [15], and antibiotic resistance genes [16,17]. This contamination poses a significant risk to human health [18], particularly when the water is used for irrigation purposes throughout the basin. Some Colombian farmers have adopted artisanal irrigation and drainage systems for their crops in Cota, Mosquera, and Funza municipalities. However, these systems may inadvertently contribute to increased bacterial loads, including pathogenic bacteria that could colonize the interior of plant tissues such as leaves, seeds, roots, stems (endophytes) and that, in addition, can alter the soil microbiome. Consequently, this situation poses a potential threat not only to lettuce crops but also to consumers [19,20]. Considering this, we propose the hypothesis that bacteria present in the irrigation water of the Bogotá River can colonize the rhizosphere of plants, such as lettuce, and subsequently migrate to their leaves. This hypothesis is supported by previous findings indicating that certain clinical pathogens could colonize both the roots and leaves of vegetables [12,20]. However, the extent to which irrigation water bacteria contribute to the bacterial diversity of the rhizosphere and endophytic community in plants, specifically lettuce, remains unclear. Therefore, the objective of this study was to assess the bacterial biodiversity in irrigation water, soil, and lettuce leaves in three municipalities located in the Bogotá savanna, aiming to identify potential sources of human pathogens associated with lettuce cultivation. We selected three municipalities based on their varying levels of contamination resulting from the discharge of wastewater from the city of Bogotá. Among these municipalities, Mosquera exhibits the highest level of contamination, followed by Funza and Cota. Two farms that utilize irrigation water from the Bogotá River were chosen from each municipality, and the taxonomic diversity of the 16S rRNA gene was analyzed in lettuce leaves, rhizospheric soil, and irrigation water for subsequent comparison.

## 2 Materials and methods

### 2.1 Study area and sampling

**Fig. 1:**
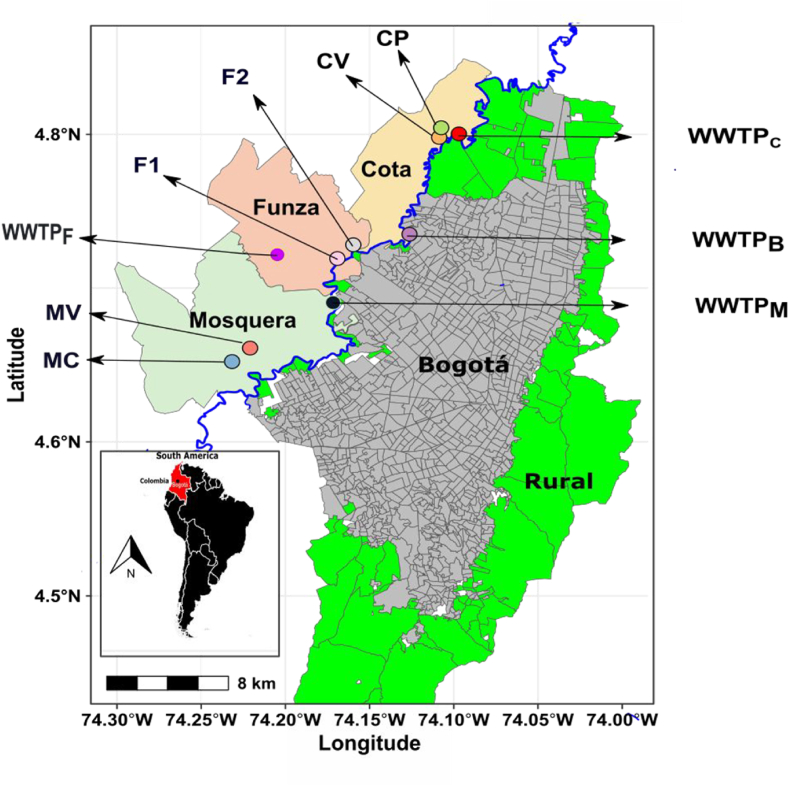
Study Area Map: The map depicts the sampling locations of farms with lettuce crops along the Bogota River in the municipalities of Cota, Funza, and Mosquera. The abbreviations used for the farms are as follows: Funza 1 = F1; Funza 2 = F2; Mosquera - La Victoria = MV; Mosquera La-Campiña = MC; Cota Variante = CV; Cota Principal = C; Wastewater treatment plants (WWTP), WWTPC = WWTP Cota; WWTPB = WWTP Bogotá; WWTPF = WWTP Funza; WWTPM = WWTP Mosquera; The blue line corresponds to Bogotá River. (For interpretation of the references to color in this figure legend, the reader is referred to the Web version of this article.)

The water, rhizospheric soil, and lettuce samples were collected during the dry season, specifically between July and August, from six farms engaged in lettuce cropping in the Cota, Funza, and Mosquera municipalities situated in the middle basin of the Bogotá River (Fig. 1) and in ascending order of anthropogenic contamination as reported by Posada-Perlaza [18]. At each farm, four integrated samples of irrigation water (15.2 L) were collected using sterile high-density polyethylene bottles and promptly transported to the laboratory. The irrigation water samples were then filtered using qualitative disc filters (BOECO Germany) to eliminate large solid residues (size >8 μm), and genetic material extraction was carried out immediately. For each farm, three integrated samples were created by combining 15 subsamples of soil/rhizosphere roots. These subsamples were collected along transects spanning approximately 25 m across the entire plot. Additionally, three integrated soil samples were obtained from each farm to assess the physical-chemical parameters. Likewise, 15 complete lettuce plants were selected to generate three integrated samples per farm. The plants were carefully wrapped in absorbent towels and packed in sealed plastic bags, along with the rhizosphere and soil samples. The collected samples were stored at 4 °C until used for analysis, all procedures and materials used met sterile conditions.

### 2.2 Physicochemical analysis of soil

An integral sample was analyzed from each of the six farms (n = 6). The following measurements were conducted to assess soil characteristics: pH and electrical conductivity were measured using a potentiometer in a soil and water mixture at a ratio of 1:1 (w/v) [21]. The exchangeable contents of Ca, K, Mg, and Na at pH 7.0 were determined through atomic absorption spectrophotometry [22]. The exchangeable acidity of the soil samples, measured at pH < 5.5, was determined using the potentiometric method [23]. The organic carbon content was measured using the Walkley-Black method [24]. The availability of P was assessed using colorimetry [25], while the S content was evaluated using UV–Vis spectrophotometric techniques [26]. Microelements (Cu, Fe, Mn, and Zn) were extracted using a modified Olsen extraction solution and measured through atomic absorption spectrophotometry [27]. Boron was extracted using monobasic phosphate (azomethine-H) [28]. The Effective Cation Exchange Capacity was calculated following the method of the Sociedad Colombiana de la Ciencia del Suelo in 1981. Soil texture was analyzed using the Bouyoucos hydrometer method [29]. The total nitrogen content was determined using the semi-micro-Kjeldahl method [30]. To assess significant differences between physicochemical parameters and sampling points, an analysis of variance was performed. The relationships between the physicochemical data and sampling points were established using correspondence analysis. These analyses were conducted using R Studio version 4.1.1.

### 2.3 DNA extraction and sequencing

DNA extraction was carried out on all environmental samples, including rhizosphere soil, irrigation water, and lettuce endophytes, using the DNeasy® PowerSoil Pro® kit following the provider's protocol with minor modifications specific to the irrigation water samples and lettuce endophytes. For the irrigation water samples, each sample was filtered through 0.22 μm polyamide filters (GE Healthcare Life Sciences Whatman™). The filters were then carefully cut using sterile scissors and placed in the DNeasy® PowerSoil Pro® bead tubes. Lettuce plant samples were collected and washed under running tap water to remove any soil particles. They were then surface sterilized with a sodium hypochlorite solution (2 %) containing 0.1 % Tween 20 for 3 min. Afterward, the samples were rinsed three times with sterile distilled water and dried using sterile paper towels (D'Amico et al., 2008). The collected samples were pooled, and the previously sterilized tissue was macerated to a fine powder in liquid nitrogen using an autoclaved mortar and pestle. Total DNA extraction was performed on the macerated samples. A 30 ng qualified DNA template and 16S rRNA V3–V4 region primers (338F: ACTCCTACGGGAGGCAGCAG; 806R: GGACTACHVGGGTWTCTAAT) were added for Polymerase Chain Reaction (PCR). All PCR products were purified using Agencourt AMPure XP beads (Beckman Coulter™), dissolved in Elution Buffer, and labeled to complete library construction. The library size and concentration were assessed using an Agilent 2100 Bioanalyzer (Agilent Technologies). Qualified libraries were sequenced on an Illumina™ HiSeq 2500 platform according to their insert size.

### 2.4 Bioinformatic and statistical analysis

The 16S rRNA raw reads sequencing data is available at the Zenodo repository (https://doi.org/10.5281/zenodo.10652573). For V3–V4 region of the 16S rRNA gene, the pair-end reads were assembled using the PEAR software [31]. Following primer removal, the sequences were filtered based on quality score (Q = 30), minimum expected error (0.1), and length. Dereplication of the sequences was performed using VSEARCH [32]. The sequences were then clustered at 100 % similarity to form zero-radius operational taxonomic units (zOTUs) using USEARCH [33]. Taxonomic classification of the sequences was carried out using the SINTAX command [34] against the SILVA database v.138. Biodiversity analysis was performed using MicrobiomeAnalyst (https://www.microbiomeanalyst.ca/). Prior to analysis, data underwent a filtering process with a minimum count threshold of four zOTUs per sample, a sample prevalence of 10 zOTUs, and low variance filtering at 10. Data normalization was performed using the cumulative sum scaling method. The alpha diversity was determined using the Shannon and Chao1 indices. Beta diversity comparison between samples and sampling sites was evaluated using the Bray-Curtis dissimilarity index. Additionally, a principal component analysis (PCA) was conducted to explore the similarity of microbial communities. To associate the physicochemical parameters with the most abundant genera detected in water, soil, and inside lettuce, Pearson correlation and principal component analysis (PCA) were conducted using R v.3.6.1.

## 3 Results

### 3.1 Physicochemical analysis of soils

**Fig. 2:**
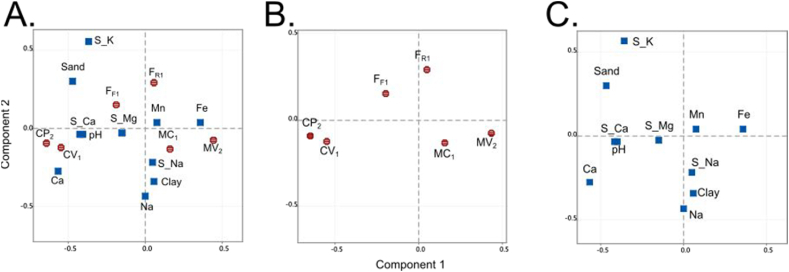
Component analysis depicting the relationship between A) Lettuce farms and physicochemical soil parameters, B) farms only, and C) physicochemical soil parameters. Blue squares: Physicochemical parameters. Red circles sampling sites. (For interpretation of the references to color in this figure legend, the reader is referred to the Web version of this article.)

The municipalities exhibited significant differences in 11 physicochemical soil parameters (Table S1). The component analysis effectively differentiated the farms based on their respective municipalities (Fig. 2). Specifically, the soils in Cota displayed elevated levels of calcium and pH. On the other hand, the soils in Mosquera exhibited higher concentrations of sodium, iron, and clay, while the soils in Funza had higher levels of sand and potassium saturation (Table S1).

### 3.2 Bacterial diversity in soil, irrigation water, and lettuce plants

**Fig. 3:**
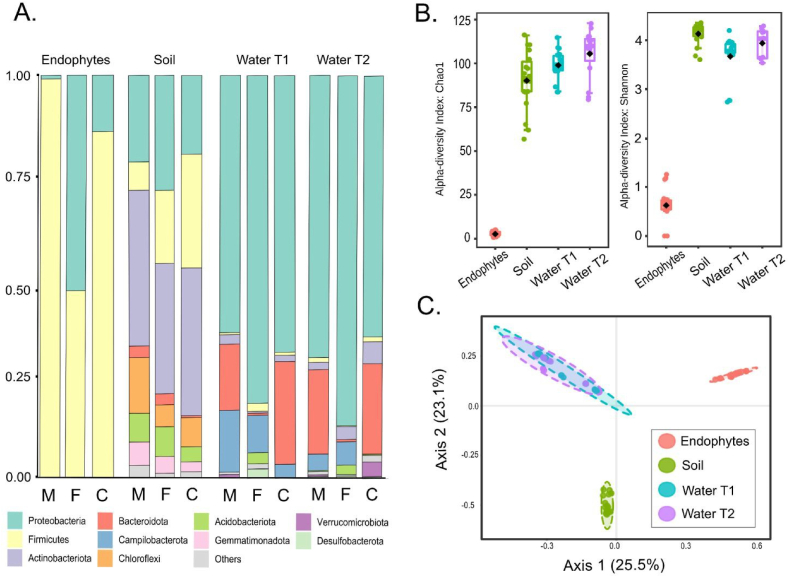
Bacterial diversity in endophyte samples from lettuce plants, lettuce rhizospheric soil, and irrigation water at one month (T1) and one week (T2) after harvest. A) Relative abundances at the phylum level for Mosquera (M), Funza (F), and Cota (C). B) Diversity indices (Chao1 and Shannon). C) Principal Component Analysis (PCA) for endophyte, soil, and water matrices.

Bacterial biodiversity was classified into three categories: endophytes, irrigation water, and soil, irrespective of their geographic origin (Fig. 3D). Endophyte samples displayed the lowest diversity indices (Fig. 3B) and were characterized by a high abundance of Firmicutes (52.8 %) and Proteobacteria (47.2 %). Soil samples exhibited the highest abundances of Actinobacteriota (34.8 %), Proteobacteria (23.4 %), and Firmicutes (15.7 %). Irrigation samples demonstrated the highest abundances of Proteobacteria (71.3 %), Bacteroidota (15.8 %), and Campylobacterota (6.2 %) (Fig. 2A). The genus *Bacillus* (zOTU2) was the only taxonomic category found across all three matrices. Only seven zOTUs were shared between endophytes and irrigation water, including four Firmicutes from the genera *Bacillus*, *Lactococcus*, and *Exiguobacterium* (2 zOTUs), and three Proteobacteria (*Aeromonas* and *Pantoea*). Similarly, only three zOTUs from the genus *Bacillus* and two from *Enterobacter* were shared between endophytes and soil. Among the Funza samples, the highest abundances and genera of potentially pathogenic endophytes were observed. However, only two zOTUs from the genus *Enterobacter* were shared between endophytic and rhizospheric bacteria. Three zOTUs from the genera *Aeromonas*, *Pantoea*, and *Exiguobacterium* were shared between endophytic and irrigation water bacteria from Cota, while only one zOTU from the genus *Aeromonas* was shared in Mosquera.

### 3.3 Diversity of bacteria in irrigation water

**Fig. 4:**
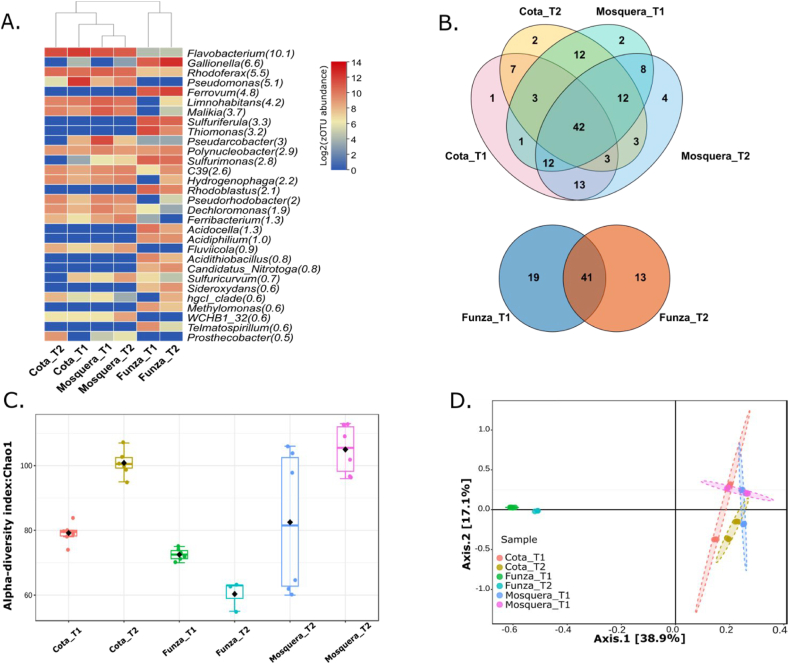
Diversity of bacteria in irrigation water samples from three municipalities. A) Relative abundance at the genus level for Mosquera, Funza, and Cota in two sampling times. B) Venn diagram at the genus level to Mosquera vs Cota and Funza 1 vs Funza 2, C) Chao1 diversity indices, D) PCA for all municipalities.

In the irrigation waters, a total of 159 genera was identified, of which 42 were common at both time 1 and time 2 for the municipalities of Cota and Mosquera. However, 10 genera were exclusive to Cota, while 14 were exclusive to Mosquera. On the other hand, in the samples from Funza, 41 genera were shared, but 19 unique genera were detected in Funza_T1 and 13 in Funza_T2 (Fig. 4B). Regarding the prevalence of genera, no significant differences were observed between Mosquera and Cota (Fig. 4D). However, the samples from Mosquera exhibited the highest bacterial diversity (Fig. 4C), followed by Cota and Funza. Additionally, certain genera were identified to harbor opportunistic pathogens for both humans and animals, such as *Pseudomonas* representing 5.1 % of the total genera (Fig. 4A).

### 3.4 Diversity of lettuce rhizospheric bacteria

**Fig. 5:**
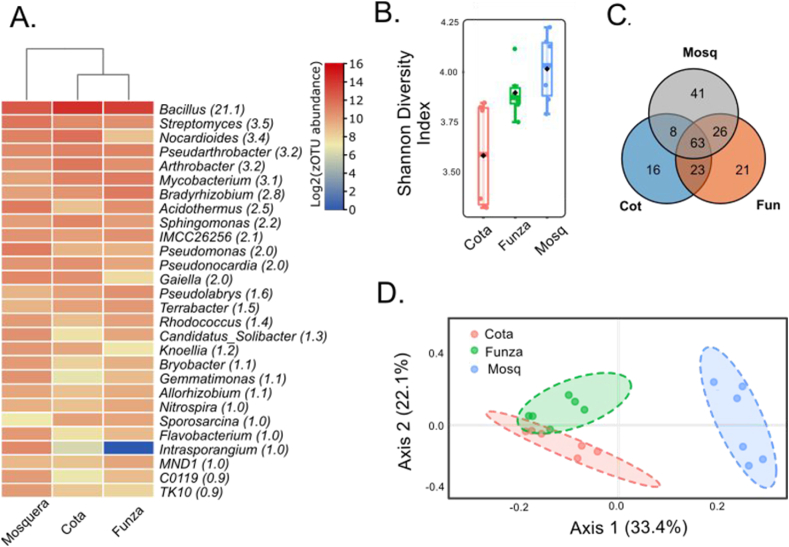
Diversity of lettuce rhizospheric bacteria in the municipalities of Mosquera, Funza, and Cota. A) Heat map displaying the most abundant genera, with relative abundance shown in parentheses. B) Shannon diversity index, C). Venn diagram illustrating the shared and unique genera among the municipalities of Cota, Funza, and Mosquera, D). PCA representing the distribution of the municipalities.

**Fig. 6:**
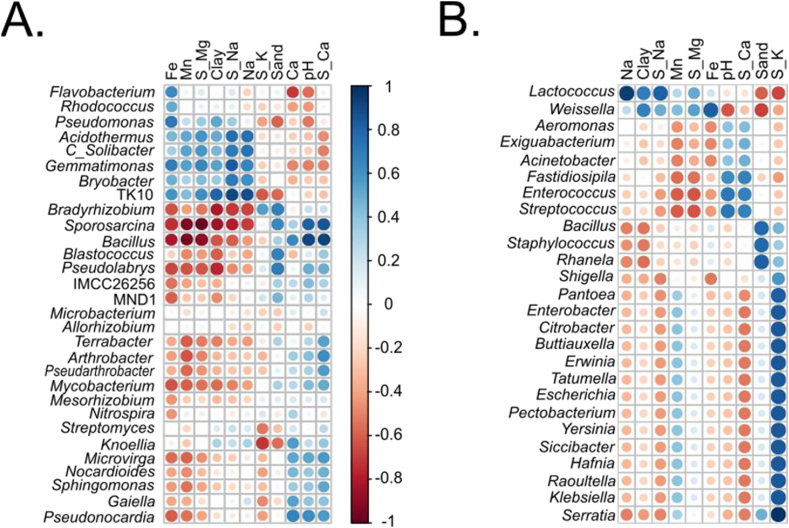
Relationship between soil physicochemical parameters and A) rhizosphere genera, and B) lettuce endophytes. S_ = Saturation.

A total of 198 genera were detected in the rhizospheric soil samples, with the most prevalent genera being *Bacillus* (21.12 %), *Streptomyces* (3.48 %), *Nocardioides* (3.36 %), *Pseudarthrobacter* (3.25 %), and *Arthrobacter* (3.20 %) (Fig. 5). Mosquera samples exhibited higher bacterial diversity compared to Funza and Cota (Fig. 5B). Exclusive genera were found for Mosquera (41), Funza (16), and Cota (21; Fig. 5C). Shared genera were observed in 63 samples (32 %), while significant differences were found in 105 genera (53 %). Beta diversity analysis indicated that Mosquera's diversity is distinct from Funza and Cota samples (Fig. 5D). Some genera, including *Mycobacterium*, *Streptomyces*, and *Nocardioides*, which have the potential to be human pathogens, were positively correlated with Ca concentrations. Similarly, *Pseudomonas*, which correlated with Fe concentrations and the percentage of clay, was also identified (Fig. 6). Additionally, genera associated with nitrogen cycling, such as *Allorhizobium, Bradyrhizobium, Rhizobium, Nitrospira*, and *Azotobacter*, were detected.

### 3.5 Diversity of endophytic bacteria

**Fig. 7:**
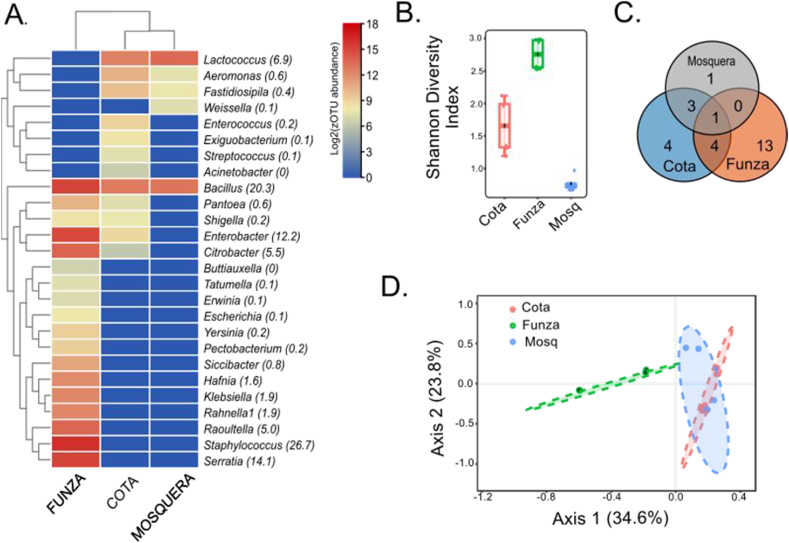
Diversity of endophytic bacteria in lettuce for the municipalities of Mosquera, Funza, and Cota: A). Heatmap of the most abundant genera. The abundance percentage is shown in parentheses, B). Shannon diversity index, C). Venn diagram at genus level, D). PCA representing the distribution of the municipalities.

A total of 26 genera of lettuce endophytic bacteria were detected. Most of these microorganisms belonged to the Proteobacteria and Firmicutes phyla. The most abundant genera were *Staphylococcus* (26.7 %; detected only in Funza), *Bacillus* (20.3 %), *Serratia* (14.1 %), and *Enterobacter* (12.2 %). The genera associated with Proteobacteria were predominantly found in Funza (50 % of the population), followed by Cota (20 %) and Mosquera (Fig. 7). The genera *Citrobacter*, *Pantoea*, and *Shigella* exhibited significant differences in abundance between Funza and Cota. *Lactococcus*, *Aeromonas*, and *Fastidiosipila* were found in Mosquera and Cota. The genus *Weissella* was only detected in Mosquera. The physicochemical parameters of the soil were found to be correlated (p-value <0.05) with different endophytic bacterial genera (Fig. 6B). *Lactococcus* and *Weissella* were favored by higher concentrations of sodium, manganese, iron, and clay. *Aeromonas, Acinetobacter, Fastidiosipila,* and others were favored by higher calcium saturation. *Shigella, Pantoea*, and *Serratia* showed a preference for higher potassium saturation. Additionally, the percentage of sand favored the presence of the genera *Bacillus, Staphylococcus*, and *Rhanella* (Fig. 6B).

## 4 Discussion

Bacterial characterization of lettuce crops, soils, and irrigation water plays a crucial role in implementing the farm-to-fork strategy, enhancing the monitoring of food production practices, inspections, and safety testing. This comprehensive approach addresses potential hazards throughout the entire food chain, from primary production to marketing. Thus, our hypothesis suggests that bacterial pathogens in lettuce may come from resident populations from irrigation water and rhizospheric soil or even endophytic bacteria. However, the diversity of endophytic bacteria cannot be solely attributed to the diversity of soil and irrigation water bacteria. Among the 163 zOTUs recorded for endophytes, only nine were shared across matrices. The limited number of shared zOTUs and the differentiation of matrices observed through beta diversity analysis (Fig. 2D) indicate that each environment is distinct and represents a highly specialized niche that is challenging for the same bacteria to colonize. However, the genus *Bacillus* was detected in all three environments, suggesting a potential ability to thrive across diverse conditions. Bacteria that establish colonization within plant tissues must overcome the plant's defense mechanisms to avoid being recognized as pathogens, implying a coevolutionary pattern between plants and bacteria [35]. Rhizobacteria can successfully colonize plant roots if they can metabolize rhizosphere exudates and establish beneficial relationships with the plant [36]. Most bacterial genera present in irrigation water are harmless to humans, as they are natural inhabitants of surface freshwater bodies, contribute to biogeochemical cycles, and participate in the degradation of various xenobiotics.

However, certain genera containing pathogenic species were detected in irrigation water at low concentrations (less than 0.1 %), such as *Pseudomonas, Flavobacterium, Serratia, Enterobacter, Klebsiella, Yersinia, Staphylococcus,* and *Streptococcus*. Consequently, the low abundance of pathogenic bacteria in environments such as water, and even in soil, would limit the opportunities to colonize the interior of lettuce plants. However, it has been reported that clinically important pathogenic bacteria can colonize the interior of vegetables, including lettuce, under controlled conditions [37,38]. One example is the human pathogen *E. coli* O157:H7, which can colonize lettuce plants. However, under field conditions, this pathogen cannot significantly increase its population on lettuce leaves, and its persistence may be linked to its ability to occupy microniches on damaged leaves [38]. This suggests that *E. coli* O157:H7 behaves as an opportunistic pathogen rather than utilizing lettuce plants as a reservoir for disease transmission. In our study, the counts for potential Enterobacterales pathogens were relatively low, with only 173 reads detected in irrigation water and 1469 reads in soil. It is worth noting that inoculation assays of pathogenic bacteria in plants are often conducted under non-field conditions, using high bacterial concentrations (1 × 10^5^ cells ml^−1^), and measuring colonization over short periods of time, typically not exceeding 24 h [38]. This limits our understanding of the pathogen's long-term persistence and multiplication throughout the crop cycle.

### 4.1 Microbial diversity in irrigation water

The Bogotá River is known for its high bacterial load and contamination by xenobiotic compounds, making it one of the most polluted rivers in the country [15,16]. Despite this, the river water is frequently used for irrigation in agricultural fields surrounding the Bogotá savanna [13,18]. Although the implemented strategy did not allow the determination of bacterial species, our study revealed that the presence of pathogenic bacteria genera in the irrigation water was relatively low, and most of the detected genera belonged to opportunistic groups that pose a low risk to human health. The risk of infection from consuming food contaminated with bacteria depends on various factors, including the type of bacteria, their concentration, the type of food, its processing and storage conditions, and the immune status of the consumer [39]. For instance, Salmonella can cause severe infections even with a few cells, while other bacteria may require a higher dose to cause illness [40]. However, we did identify some pathogenic species such as *E. meningoseptica*, which can cause infections in newborns and immunocompromised individuals. Additionally, certain species of *Pseudomonas*, including *P. aeruginosa*, are known to be pathogenic to both humans and animals, causing opportunistic infections particularly in individuals with compromised immune systems. It is noteworthy that many bacterial genera found in the irrigation water samples play significant roles in biogeochemical cycles such as sulfur, iron, carbon, and nitrogen cycles, making them of great interest in biotechnology applications [41]. In previous studies conducted in 2015 and 2017 in the same irrigation district, a high population of coliforms, around 1 × 10^7^ CFU mL^−1^, as well as pathogens such as *E. coli* and *Pseudomonas* sp., were reported [42,43]. In contrast, our samples exhibited a significantly lower number of total coliforms, at 1 × 10^3^ CFU mL^−1^ (data no shown).

### 4.2 Biodiversity of rhizospheric bacteria in lettuce crops

The soil could not be considered a source of potentially pathogenic bacteria that colonize the interior of lettuce plants, since the main pathogens, such as *Mycobacterium*, *Streptomyces*, *Nocardioides* and *Pseudomonas*, were not identified as endophytes (Fig. 5). However, the genus *Bacillus* was found to be the most abundant both in the lettuce rhizosphere and inside the plants, while it was less abundant in irrigation water (Fig. 4, Fig. 5, Fig. 6). *Bacillus* has been recognized as an opportunistic human pathogen [44,45] and as a plant growth promoter through mechanisms such as phosphorus solubilization, nitrogen fixation, induction of systemic resistance, and control of phytopathogens (Ferreira et al., 2011; [46,47]. *Bacillus* establishes itself in the rhizosphere of lettuce plants, particularly in the primary roots, root hairs, and tips, as well as in adjacent border cells [48]. This colonization enables *Bacillus* to stimulate the production of secondary metabolites, activate the plant's immune system against plant pathogens, and facilitate nutrient absorption in the plant [49,50].

Streptomyces, the second most abundant genus in the soil, plays a functional role in nitrogen fixation [51] and produces antimicrobial substances [52]. It is also known to cause phytopathological diseases such as scab disease in potatoes and soft rot in vegetables [53]. The detection of genera like Mycobacterium is of significant concern, as they are associated with foodborne mycobacterial diseases [54]. Nontuberculous mycobacteria, such as *M. fortuitum*, *M. bourgelatii*, *M. goodii*, *M. intracellulare*, *M. aromaticivorans*, and *M. avium*, which were presumptively detected, are opportunistic pathogens that pose a higher risk to certain groups, particularly those with underlying lung disease or weakened immune systems. While these pathogens are not typically transmitted from person to person [55], *M. avium* has been detected in apple juice, salads, leeks, lettuce, mushrooms, and other vegetables. It is worth noting that mycobacteria are persistent in soil, manure, and various ecosystems [54].

Similarly, opportunistic pathogenic genera such as *Pseudomonas, Streptomyces*, and *Nocardioides* pose a constant risk to susceptible animal species and immunocompromised humans. These bacteria are associated with a wide range of diseases and can cause severe pneumonia [56], bloodstream infections [57], urinary tract infections, surgical site infections [58], and other infections. Additionally, opportunistic infections and microbiome alterations might result in negative effects on host immune responses [59]. Furthermore, these bacteria's pathogenic role in human health is closely linked to the emergence of antimicrobial resistance. Although *Pseudomonas* is not considered a significant foodborne pathogen, several studies have reported significantly higher rates of aminoglycoside resistance in *Pseudomonas* isolates from fruit vegetables, tubers, and salads [60,61]. However, other genera such as *Bradyrhizobium*, *Rhizobium*, *Nitrospira*, *Azotobacter*, *Arthrobacter*, *Bacillus*, *Burkholderia*, *Enterobacter*, and *Pseudomonas* establish beneficial relationships with plants. They contribute to nitrogen fixation, solubilize phosphorus, produce siderophores, synthesize plant growth regulators, and act as biocontrol agents against plant pathogens [[62], [63], [64]].

### 4.3 Biodiversity of endophytes associated with lettuce plants

The lettuce's highest values of the abundance of endophytic microorganisms were observed in the genus *Staphylococcus* was found to have the highest abundance of endophytes in lettuce (Fig. 7). It is known that some species of this genus can cause a variety of infections, including skin infections, bacteremia, bone infections, endocarditis, food poisoning, pneumonia, and toxic shock syndrome. It is important to note that the presence of *Staphylococcus* is associated with a higher horizontal transmission of antibiotic resistance genes through mobile genetic elements [20]. Additionally, this genus has been reported in lettuce salads in Brazil [65] and is correlated with foodborne diseases due to toxin production [66,67]. The genus *Bacillus* was detected in abundance (20.3 %) in all three sampling sites. Species within this genus, such as *B. cereus* and *B. mycoides*, have been detected in lettuce plants [68]. The presumptive detection of *B. cereus* is of concern as this species is associated with two types of foodborne illnesses: the diarrheal form and the emetic form. This species is highly persistent due to its ability to form resistant spores and its capacity to adhere and form biofilms on the surface of lettuce leaves [69]. The family Enterobacteriaceae was found to be abundant, and the presence of certain species from genera such as *Escherichia*, *Klebsiella*, *Enterobacter*, and *Citrobacter* is considered indicative of fecal contamination by coliforms. In areas like Funza, diverse Enterobacteria from genera such as *Serratia*, *Enterobacter*, *Citrobacter*, *Raoultella*, *Klebsiella*, *Rahnella*, *Hafnia*, *Siccibacter*, *Pantoea*, *Yersinia*, *Shigella*, *Escherichia*, and *Erwinia* were detected (Fig. 7). Our findings suggest that the presence of enterobacterial populations in Funza could be influenced by soil salinity. This is supported by a positive correlation observed between enterobacteria and the percentage of K saturation (Fig. 6B), as well as the higher electrical conductivity values (3.27 dS/m) found in this area. On the other hand, the dominant genera in the rhizospheric soil, such as *Bacillus* and *Nocardioides*, showed a negative response to salinity (Fig. 6A). Salinity is widely recognized as a key factor driving changes in the composition of bacterial communities [70]. Furthermore, it is possible that the composition of the endophytic community is a plant-driven mechanism to select bacteria that assist in mitigating salt stress [71].

Enterobacterales are commonly found in the intestines of humans and animals but can also act as opportunistic pathogens [72]. The pathogenicity of enterobacteria is determined by various factors, including their capacity for adherence and invasion, resistance to host defenses, toxin production, acquisition of resistance genes, and interaction with the intestinal microbiota [73]. This pathogenicity can vary among specific species and strains, as well as in relation to the host. For instance, enterobacteria can produce toxins such as Shiga toxin, thermostable and thermolabile toxins, and cytotoxic toxins, which contribute to pathogenicity [74]. Regarding enterobacteria present in food, Ye et al. [75] reported a significant percentage of food-isolated Enterobacteriaceae showing resistance to cephalosporins, as well as phenotypes of extended-spectrum beta-lactamase. They also found a high percentage of strains resistant to multiple antibiotics. These findings highlight the importance of considering food, such as raw vegetables, as potential reservoirs for resistance genes and their possible transmission through the food chain. Similar studies conducted by Song [76] and Pintor-Cora [77] support these conclusions by isolating beta-lactamase-producing enterobacteria from raw vegetables.

The lack of correlation observed between lettuce endophytes, soil bacteria, and irrigation water in this study may be attributed to other factors that were not evaluated. For example, it is known that plant microbiota can be transmitted through seeds [78], and specific bacteria from the Enterobacteriaceae family can be selected to establish beneficial relationships that enhance plant growth [36,50,79]. Additionally, the composition of endophytes may be influenced by cumulative effects, where contributions from dynamic environments such as irrigation water and soil may not be readily apparent. For example, some detected pathogenic endophytes could have been acquired in early irrigation of the crop with waters with a higher microbial load of pathogens. It is worth noting that soil microbial diversity can vary with the plant's phenological stage [80], soil fertility type, and crop management practices [81]. While the endophyte samples in our study showed a high representation of Proteobacteria, particularly Enterobacterales, we cannot conclude that they pose a risk to human health. The pathogenicity of bacteria depends on factors such as bacterial concentration, virulence factors, and the frequency of exposure. However, it is worth mentioning that potentially pathogenic genera are commonly found in lettuce endophyte microbiomes even in the absence of exposure to contaminated irrigation water. To assess the pathogenic potential of these endophytic pathogens, we are currently characterizing the genomes of cultivable lettuce endophytes and conducting metagenomic sequencing of total lettuce endophyte samples to detect virulence factors such as toxins, antibiotic resistance genes, and pathogenicity islands, as well as to improve taxonomic identification. To obtain a more accurate estimation of risk, it is recommended to monitor the presence of endophytes in a larger number of lettuce-cultivated areas and expand this type of study to other leafy vegetables that may harbor potential human pathogens. This broader approach will help provide a comprehensive assessment of the risks associated with endophytic bacteria in the context of food safety.

## 5 Conclusions

While potentially pathogenic bacteria genera were found in both the irrigation water and soil, there is no evidence to suggest that they were the primary contributors to the endophytic bacterial community in lettuce. Only a few operational taxonomic units were shared across different sample types. However, the genus *Bacillus* was consistently abundant in all three environments, indicating its potential to thrive in diverse conditions. The composition of the endophytic microbiome differed significantly from that of the soil and irrigation water, suggesting the presence of specific selection pressures within the plant's ecosystem. It is important to note that the dominance of Proteobacteria, particularly Enterobacterales, in the endophytic community does not necessarily indicate pathogenicity. This is due to their low abundances, taxonomy limited to presumptive species, and unknown virulence factors. Additionally, many of these enterobacteria have been reported as natural endophytes of lettuce plants. Other factors remain to be assessed, such as spread through seeds, variations in bacterial composition of irrigation water during non-sampling periods, or dynamic changes in population within the evaluated matrices. These findings highlight the complexity of the bacterial community within lettuce plants, requiring further investigation to determine the associated risks to food safety. Conducting a larger-scale study that monitors endophytes in different regions and crops would provide a more comprehensive evaluation of the risks posed by these bacterial communities in terms of public health. Overall, this study contributes valuable insights into the ecology of bacterial communities associated with lettuce cultivation and emphasizes the importance of monitoring all stages of lettuce production under the farm-to-fork strategy to mitigate public health risks and ensure food safety.

## Funding

The authors gratefully acknowledge Universidad Antonio Nariño, Universidad de Antioquia, Universidad del Valle, and 10.13039/100022965MINCIENCIAS COLOMBIA for their funding through the program PRO- CEC-AGUA, contract 80740-173-2021 with code 111585269594.

## CRediT authorship contribution statement

**Rodrigo A. Echeverry-Gallego:** Conceptualization, Data curation, Formal analysis, Investigation, Methodology, Software, Visualization, Writing – original draft, Writing – review & editing. **Diana Martínez-Pachón:** Conceptualization, Formal analysis, Investigation, Methodology, Supervision, Writing – original draft, Writing – review & editing. **Nelson Enrique Arenas:** Investigation, Methodology, Writing – original draft, Writing – review & editing. **Diego C Franco:** Methodology, Writing – original draft, Writing – review & editing. **Alejandro Moncayo-Lasso:** Conceptualization, Formal analysis, Investigation, Methodology, Supervision, Writing – original draft, Writing – review & editing. **Javier Vanegas:** Conceptualization, Data curation, Formal analysis, Investigation, Methodology, Supervision, Writing – original draft, Writing – review & editing.

## Declaration of competing interest

The authors declare that they have no known competing financial interests or personal relationships that could have appeared to influence the work reported in this paper.
