# Supplementary material for: Characterization of bacterial diversity in rhizospheric soils, irrigation water, and lettuce crops in municipalities near the Bogotá river, Colombia
Source: Heliyon. 2024 Aug 6;10(16):e35909. doi: 10.1016/j.heliyon.2024.e35909 (PMC11369436; doi:10.1016/j.heliyon.2024.e35909)
Supplement: Multimedia component 1 [file mmc1.docx]

**Supplementary material**

**Characterization of bacterial diversity in rhizospheric soils, irrigation water, and lettuce crops in municipalities near the Bogotá River, Colombia.**

Rodrigo A. Echeverry-Gallego^1,2^, Diana Martínez-Pachón^1^, Nelson Enrique Arenas^1,3^, Diego Castillo Franco^4^, Alejandro Moncayo-Lasso^1^, Javier Vanegas^1^

1 Grupo de Investigación en Ciencias Biológicas y Químicas, Facultad de Ciencias, Universidad Antonio Nariño, Bogotá DC, Colombia.

2 Doctorado en Ciencia Aplicada, Universidad Antonio Nariño, Bogotá DC, Colombia.

3 Facultad de Medicina, Universidad de Cartagena. Cartagena, Colombia.

4 Institute of Environmental Sciences, Faculty of Biology, Jagiellonian University, Krakow, Poland.

Corresponding author Javier Vanegas [javane](mailto:javanegas100@uan.edu.co)[gas100@uan.edu.co](mailto:gas100@uan.edu.co)

https://orcid.org/0000-0003-2257-3056

Table S1. Main physicochemical parameters of lettuce farm soil

| ID | CV | CP | F1R | F1F | M1C | M2V |  | Cota | Funza | Mosquera | p-value |
| --- | --- | --- | --- | --- | --- | --- | --- | --- | --- | --- | --- |
| Sulfur (S) available | 29,8 | 37,0 | 64,7 | 77,1 | 70,1 | 31,0 | 51,6 | 33,4 | 70,9 | 50,6 | 0,2 |
| Boron (B) available | 0,7 | 0,7 | 1,3 | 0,9 | 0,9 | 0,7 | 0,8 | 0,7 | 1,1 | 0,8 | 0,3 |
| Calcium (Ca) available | 27,0 | 23,8 | 8,3 | 16,0 | 20,2 | 14,7 | 18,3 | 25,4 | 12,1 | 17,5 | 0,1 |
| Effective Cation Interchange Capacity (CICE) | 28,4 | 26,2 | 10,2 | 22,8 | 27,5 | 19,7 | 22,5 | 27,3 | 16,5 | 23,6 | 0,3 |
| Organic Carbon (CO) | 5,7 | 5,0 | 2,9 | 5,6 | 3,1 | 3,1 | 4,2 | 5,3 | 4,2 | 3,1 | 0,3 |
| Copper (Cu) olsen Available | 2,3 | 4,3 | 4,3 | 2,6 | 4,0 | 6,9 | 4,1 | 3,3 | 3,5 | 5,5 | 0,4 |
| Electrical conductivity (EC) (1:5) | 1,3 | 1,6 | 2,2 | 4,3 | 1,7 | 1,4 | 2,1 | 1,4 | 3,3 | 1,6 | 0,2 |
| Phosphorus (P) Available (Bray II) | 185,7 | 194,9 | 108,7 | 261,5 | 258,0 | 89,2 | 183,0 | 190,3 | 185,1 | 173,6 | 1,0 |
| Iron (Fe) olsen Available | 64,5 | 45,6 | 243,1 | 123,8 | 247,7 | 448,3 | 195,5 | 55,0 | 183,5 | 348,0 | 0,1 |
| Magnesium (Mg) Available | 3,2 | 3,5 | 1,4 | 3,9 | 5,6 | 3,9 | 3,6 | 3,3 | 2,7 | 4,8 | 0,3 |
| Manganese (Mn) olsen Available | 2,9 | 3,9 | 7,0 | 12,5 | 12,9 | 12,8 | 8,7 | 3,4 | 9,7 | 12,8 | 0,1 |
| Organic Matter (OM) | 8,8 | 7,8 | 4,6 | 9,6 | 5,1 | 5,2 | 6,9 | 8,3 | 7,1 | 5,2 | 0,4 |
| pH (1:2.5) | 6,9 | 6,6 | 6,0 | 5,9 | 6,1 | 5,5 | 6,2 | 6,8 | 5,9 | 5,8 | 0,1 |
| Clay percentage (% Ar) | 22,0 | 19,5 | 10,0 | 21,7 | 50,0 | 55,4 | 29,7 | 20,7 | 15,8 | 52,7 | 0,0 |
| Sand percentage (% A) | 37,0 | 39,9 | 64,1 | 43,6 | 28,9 | 15,4 | 38,1 | 38,5 | 53,8 | 22,1 | 0,1 |
| Silt percentage (%L) | 41,1 | 40,6 | 26,0 | 34,7 | 21,2 | 29,2 | 32,1 | 40,8 | 30,3 | 25,2 | 0,1 |
| Potassium (K) Available | 0,9 | 0,9 | 0,8 | 2,7 | 0,8 | 0,4 | 1,1 | 0,9 | 1,8 | 0,6 | 0,4 |
| Calcium saturation | 95,0 | 91,0 | 82,0 | 70,0 | 73,0 | 75,0 | 81,0 | 93,0 | 76,0 | 74,0 | 0,1 |
| Magnesium saturation | 11,0 | 13,0 | 14,0 | 17,0 | 20,0 | 20,0 | 15,8 | 12,0 | 15,5 | 20,0 | 0,0 |
| Potassium saturation | 3,0 | 3,0 | 8,0 | 12,0 | 3,0 | 2,0 | 5,2 | 3,0 | 10,0 | 2,5 | 0,0 |
| Sodium saturation | 2,0 | 3,0 | 3,0 | 2,0 | 7,0 | 6,0 | 3,8 | 2,5 | 2,5 | 6,5 | 0,0 |
| Sodium (Na) Available | 0,6 | 0,8 | 0,3 | 0,5 | 2,1 | 1,2 | 0,9 | 0,7 | 0,4 | 1,6 | 0,1 |
| Zinc (Zn) olsen Available | 14,1 | 17,0 | 13,4 | 19,4 | 19,5 | 21,9 | 17,5 | 15,5 | 16,4 | 20,7 | 0,3 |
